# Commentary: A tale of many cities in one: the Pelotas (Brazil) Birth Cohorts, 1982–2015

**DOI:** 10.1093/ije/dyy214

**Published:** 2019-03-18

**Authors:** Fernando C Barros, Cesar G Victora

**Affiliations:** 1 Catholic University of Pelotas; 2 Federal University of Pelotas, Pelotas, Brazil

The nine articles in this Supplement present the main results from over three decades of epidemiological research in the city of Pelotas in Southern Brazil. Our first perinatal study was inspired by the British Births study of 1970, to which we were exposed as young trainees in the UK. With the strong support of Prof. Patrick Vaughan from the London School of Hygiene and Tropical Medicine, who supervised our doctoral degrees, we obtained international funding from Canada (International Development Research Center) and the UK (Overseas Development Administration) to launch the 1982 Pelotas Birth Cohort study. Since then, a new cohort has been launched every 11 years. Over 20 000 subjects, in our city of 340 000 inhabitants, are being followed up from birth. These datasets allow not only analyses of life course epidemiology within a given cohort, but also panel type, secular trend analyses in which individuals of similar age in the four cohorts are compared.

In the Supplement, we have focused on analyses that are primarily descriptive of the health and nutrition conditions of women who gave birth, and of infants who were born, in the years 1982, 1993, 2004 and 2015. We have a special focus on socioeconomic and ethnic group inequalities, and on how these evolved over time. Our first analyses of the 1982 cohort documented the abysmal social and ethnic gaps in health, which paralleled the economic inequalities that have defined Brazilian society since early colonization and slavery. Indeed, our first book came out in 1988 with the title ‘*The Epidemiology of Inequality*’, describing differences between rich and poor mothers and children belonging to the 1982 Birth Cohort.^1^

In the 33 years that elapsed between the first and the fourth cohorts, Brazil in general and Pelotas in particular experienced major changes in all aspects of life and society—socioeconomic development, culture, moral values, physical environment, technology, demography, nutrition, health care systems—all of which impacted directly on the lives of the Birth Cohort populations. Starting off as slightly more developed than Brazil as a whole, Pelotas became relatively poorer: the city was 9% above the national gross development product per capita in 1982, and is now 26% below.^2^ The decline was largely due to the bankruptcy of the city’s main economic activity in the 1980s—the canned fruit and food industries—due to the opening of the national market to cheap, subsidized foreign products in the 1990s. The social history of Pelotas is also affected by the fact that it is located in an area of large plantations and cattle farms with few landholders and many impoverished labourers, and by the forced immigration of African slaves in the 19th century to work on the salted meat (or jerky) industry.

The accompanying articles document major changes in several aspects of the health and nutrition of women and children. Table 1 summarizes the observed changes in six major sets of indicators: the profile of women giving birth; health care during pregnancy, delivery and infancy; maternal nutrition; newborn health; fetal and infant mortality and morbidity; and nutrition during infancy. In the table, we have used terminology such as ‘increased’, ‘decreased’ or ‘stable’, based on the trends, confidence intervals and *P*-values reported in the nine preceding articles. We also report on trends among ‘rich’ (top two income quintiles) and ‘poor’ (bottom two income quintiles)^2^^,^^11^ women and children, and taking into account the evolution of the indicators of absolute (the slope index) and relative (the concentration index) inequalities. We focus on changes between the two extreme years, 1982 and 2015; most changes took place gradually over time, and those for which there was evidence on non-linearity are described in the individual articles in the Supplement.

**Table 1.** Classification of health indicators according to time trends in levels and inequalities. Pelotas, Brazil, 1982–2015

**Group**^a^	Health indicators (reference)	Trends	Inequality pattern (group with higher frequencies)		Changes in income-related inequalities over time	
		Values in 2015 compared with 1982	1982	2015	Absolute inequality	Relative inequality
	Sociodemographic					
A	Maternal schooling^2^	Increased	Rich	Rich	Reduced	Stable
	Environmental					
A	Household appliances^2^	Increased	Rich	No clear pattern	Reduced	Reduced
A	Access to treated water^2^	Increased	Rich	No clear pattern	Reduced	Reduced
	Maternal nutrition					
A	Maternal height^3^	Increased	Rich	Rich	Reduced	Reduced
A	Maternal underweight^3^	Decreased	Poor	Poor	Reduced	Reduced
D	Maternal overweight^3^	Increased	Middle class	Middle class	Stable	Stable
D	Weight gain in pregnancy^3^	Increased	Rich	No clear pattern	Reduced	Reduced
	Reproductive history					
B	Maternal age <20 years^2^	Decreased	Poor	Poor	Increased	Increased
C	Maternal age >35 years^4^	Increased	Rich	Rich	Increased	Increased
B	Parity >0^4^	Decreased	Poor	Poor	Increased	Increased
B	Short birth interval^4^	Decreased	Poor	Poor	Increased	Increased
	Newborn health and nutrition					
E	Low birthweight^5^	Stable	Poor	Poor	Reduced	Reduced
E	ICU stay after birth^6^	Increased	No clear pattern*	No clear pattern	Stable	Stable
C	Multiple births^4^	Increased	No clear pattern	Rich	Increased	Increased
	Pregnancy and delivery care					
A	Antenatal care >5 visits^7^	Increased	Rich	Rich	Reduced	Reduced
C	Caesarean sections^7^	Increased	Rich	Rich	Increased	Reduced
	Mortality and hospital morbidity					
A	Fetal mortality^8^	Decreased	Poor	Poor	Reduced	Stable
E	Neonatal mortality^8^	Decreased	Poor	Poor	Stable	Stable
A	Infant mortality^8^	Decreased	Poor	Poor	Reduced	Stable
A	Hospital admissions^6^	Decreased	Poor	Poor	Reduced	Reduced
	Breastfeeding					
B	Breastfeeding at 12 months^9^	Increased	Poor	No clear pattern	Reduced	Reduced
B	Exclusive BF at 3 months^9^	Increased	Rich*	Rich	Increased	Reduced
	Child nutrition (12 months)					
A	Stunting^10^	Decreased	Poor	Poor	Reduced	Reduced
A	Wasting^10^	Stable	Poor	No clear pattern	Reduced	Reduced
D	Overweight^10^	Increased	Rich	Poor	Reduced	Reduced

ICU, intensive care unit; BF, breastfeeding.

a Group A: indicators that improved for the whole population and also showed faster progress among the poor, thus leading to reduced inequalities, at least in absolute terms. Group B: indicators with overall improvement, but for which social inequalities increased due to slower progress among the poor. Group C: indicators whose overall prevalence increased over time, mostly due to rapid increases among the rich. Group D: indicators related to the nutrition transition, which showed major increases over time. Group E: indicators that did not fit into any of previous categories.

The profiles of women giving birth changed markedly. The total number of births fell from 6011 in 1982 to 4329 in 2015, in spite of a near 50% increase in the city’s population; the birth rate fell from 23 to 13 births per thousand in this period. Fertility reduction was most marked among White mothers, whereas the number of births to women with Black or Brown skin colour remained stable in the four cohorts.^2^ On the other hand, the proportion of children born to poor families and to uneducated mothers decreased substantially, as the average schooling went up from 6.5 to 9.8 years.^2^ The proportion of adolescent mothers remained stable, but there was a marked increase in mothers aged 35 years or older.^4^ Half of all mothers are now primiparae, compared with 39% in 1982, and birth intervals became substantially longer.^4^ These findings show a marked demographic transition coupled with improved education and income, in spite of Pelotas having lost ground compared with more rapid changes in the rest of the country.

Health care also changed markedly. Whereas in 1982, 6% of the women had no health care coverage and gave birth in charity hospitals, in 1988 a national health service was created by the national constitution approved at the end of a long period of military dictatorship. Antenatal care improved in quantitative terms, with an average of over eight consultations per woman,^7^ but at the same time a massive epidemic of caesarean sections took place. In an article reporting on the caesarean section rates in 1982,^12^ we used the term ‘epidemic’ to describe the 28% rate; little did we know that 33 years later the rate would increase to 65%. Caesarean sections are concentrated among women with high socioeconomic position, reaching 85% of all births to these women in 2015.^7^ Scheduled caesarean sections, when the fetus is estimated to have reached 38 weeks of gestation, are the rule in this group.

The nutritional status of the mothers changed markedly. Low stature and underweight at the beginning of pregnancy were reduced, but 47% of the women were overweight or obese at conception, compared with 22% in 1982. There was also a sharp increase in women who gained more weight than recommended during pregnancy.^3^

The incredibly high caesarean section rate is likely to have contributed to the stabilization of low birthweight prevalence at around 10% in the last three cohorts. Birthweights would be expected to increase in light of better maternal nutrition, improved socioeconomic status and increased access to health care, but their stability signals that other factors evolved in an opposite direction. Analyses of time trends in preterm births are affected by changes in the way gestational age was assessed, but nevertheless there is evidence of a major increase in prevalence, which is in agreement with other Brazilian studies.^5^

Fetal, neonatal and infant mortality rates decreased markedly. Deaths due to diarrhoea and other infections showed the fastest decline.^8^ Hospital admissions also declined, again mostly due to reductions in infectious diseases. In contrast, admission to neonatal intensive care units increased, following the increase in availability of these beds and in the number of preterm births.^6^

Last, the durations of exclusive and any breastfeeding increased sharply.^9^ The prevalence of stunting at 12 months of age fell, whereas the prevalence of overweight more than doubled.^10^

We have attempted to summarize the evolution of maternal and child indicators over time, by taking into account the levels, confidence intervals and *P*-values for overall trends and inequality indices reported in this Supplement’s nine preceding articles. We were able to identify five groups of indicators (Table 1).

Group A includes indicators that improved for the whole population and also showed faster progress among the poor, thus leading to reduced inequalities, at least in absolute terms. These include maternal schooling, water supply and availability of household appliances, maternal height and underweight, antenatal care, fetal and infant mortality, hospital admissions and infant undernutrition.

Group B also includes indicators with overall improvement, but for which social inequalities increased due to slower progress among the poor. This was the case for three indicators related to reproductive history: parity, short birth intervals and teenage pregnancies. This group also includes the two breastfeeding indicators. Exclusive breastfeeding at 3 months started near zero and increased more rapidly among the rich than among the poor; and inequalities no longer exist in continued breastfeeding at 12 months, which was more common among the poor in 1982. These trends likely reflecting faster uptake by the rich of promotion efforts.^13^

Group C includes three indicators associated with higher risk for women and children. These are unnecessary caesarean sections, births to women aged 35 years or older and multiple births. Their overall prevalence increased over time, mostly due to rapid increases among the rich.

Group D includes indicators related to the nutrition transition, which showed major increases over time. Pre-pregnancy overweight and obesity were most common among women in the intermediate income groups throughout the 33-year period. Excessive gain during pregnancy and overweight prevalence at 12 months both increased, particularly among the poor, so that the initial finding of higher prevalence among the rich was obliterated or even reversed over time, as was the case for child overweight.

Last, three indicators did not fit into any of these categories. Low birthweight remained stable over time, but disparities were reduced because of increased prevalence among the rich. Neonatal mortality rates fell over time for all groups, but inequalities remained stable with higher rates among the poor. Neonatal intensive care admissions increased over time, but there are no clear disparities according to income, although the higher fetal and neonatal mortality rates among the poor would suggest that there should be fewer admissions among the better-off.

A comprehensive review of maternal and child health trends in Brazil, covering the period from the 1980s to 2010,^14^ showed that the changes observed in the four Pelotas cohorts are in line with the what was observed in analyses of secondary national data. The review suggested that the observed improvements were likely due: to positive changes in the social determinants of health, with consequent reduction in extreme poverty and improvements in women’s education; to vertical programmes in the 1980s and 1990s against infectious diseases (diarrhoea and pneumonia control programmes, immunization programmes and breastfeeding promotion); to the creation of the national health service in 1988, with universal access to health care and deployment of primary health care teams in rural and slum areas, contributing to reduced fertility and child mortality; and finally to progress in other sectors such as water and sanitation, education, transportation, communications and—in particular—cash transfer programmes.^14^ The review also noted the marked increase in preterm deliveries, caesarean sections and overweight or obesity among women and children.

Regarding ethnic inequalities, we applied the official, widely-used Brazilian classification based on self-reported skin colour, with three main groups: White, Brown (usually mixed European and African ancestry) and Black.^2^^,^^15^ The proportion of births by Brown and Black mothers increased from 18% to 28% during the study period, which is likely due to differential fertility as well as to affirmative actions which mean that more women are self-reporting to be Black or Brown.^15^ Health differentials according to skin colour closely followed those reported above, using stratification by income groups. This was expected, given the longlasting heritage from slavery with the consequent association between poverty and ethnicity.^2^ Nevertheless, some results merit discussion. The prevalence of adolescent pregnancy was higher among Black and Brown mothers, remaining stable over time at around 20%, well above that for White mothers. In addition, though the overall rate of late fetal mortality fell by half in the city, it remained stable at around 20 deaths per 1000 births among Black mothers.^8^ The only health indicator for which Black mothers and children had an advantage over Whites was breastfeeding at 12 months of age.^9^

The present series allows a more granular understanding of these trends, due to our ability to report on a large number of primarily collected indicators that are not available from secondary data, and especially the ability to disaggregate overall trends by socioeconomic position and ethnicity. We show that for most indicators, overall progress was accompanied by—and in fact due to—a reduction in inequalities, a win-win combination that countries should strive for. However, there were several exceptions, including indicators for which progress was accompanied by an exacerbation of inequalities, and others for which a reduction in inequalities was due to worsening situation of the poor (as for overweight) or of the rich (as for low birthweight).

By carrying out four cohort studies in the same city over more than 30 years, we have learned several lessons for other similar studies. These include: the importance of having population-based samples at the start, reflecting the full spectrum of socioeconomic and environmental conditions; the importance of using strategies to minimize losses to follow-up through involvement of the cohort members, feedback on main findings and considerate handling by the study team; and the need to adapt the topics under study to the epidemiological and nutritional transitions faced by Brazilian society.

The profound social inequalities expressed in our health indicators show that instead of being a single, homogeneous urban area, Pelotas is indeed a city made up of many cities, often with several-fold differences in health indicators according to the socioeconomic position of the women and their children, with dynamic changes over time. We are deeply indebted to the over 20 000 subjects and to their families who inhabit these many cities; without their continued support we could never have achieved the low rates of non-response that characterize our cohorts.^2^

Our analyses confirm the importance of looking beyond aggregated data, in order to understand the levels and trends in health inequalities, and propose ways in which these can be overcome. We can only hope that when the 2026 cohort takes place—as it surely will—most if not all of the challenges reported here will have been tackled.

**Conflict of interest:** None declared.
